# LOXL2 Inhibition Paves the Way for Macrophage-Mediated Collagen Degradation in Liver Fibrosis

**DOI:** 10.3389/fimmu.2020.00480

**Published:** 2020-03-31

**Authors:** Mordehay Klepfish, Tamar Gross, Milena Vugman, Nikolaos A. Afratis, Sapir Havusha-Laufer, Eli Brazowski, Inna Solomonov, Chen Varol, Irit Sagi

**Affiliations:** ^1^Department of Biological Regulation, Weizmann Institute of Science, Rehovot, Israel; ^2^Research Center for Digestive Tract and Liver Diseases, Tel Aviv Sourasky Medical Center, Tel Aviv-Yafo, Israel; ^3^Department of Clinical Microbiology and Immunology, Sackler Faculty of Medicine, Tel Aviv University, Tel Aviv-Yafo, Israel

**Keywords:** liver macrophages, lysyl oxidase like 2 (LOXL2), liver fibrosis, matrix metalloproteinases (MMPs), matrix metalloproteinase-14 (MMP-14), monocyte-derived macrophages

## Abstract

Liver fibrosis is characterized by the excessive accumulation of extracellular matrix (ECM) proteins and enzymes, especially fibrillary collagens, and represents a major cause of morbidity and mortality worldwide. Lysyl oxidases (LOXs) drive covalent crosslinking of collagen fibers, thereby promoting stabilization and accumulation of liver fibrosis while limiting its resolution. Here we show in a carbon tetrachloride (CCl_4_)-induced liver fibrosis murine model that treatment with a novel anti-lysyl oxidase like 2 (LOXL2) neutralizing antibody, which targets extracellular LOXL2, significantly improves fibrosis resolution. LOXL2 inhibition following the onset of fibrosis accelerated and augmented collagen degradation. This was accompanied by increased localization of reparative monocyte-derived macrophages (MoMFs) in the proximity of fibrotic fibers and their representation in the liver. These cells secreted collagenolytic matrix metalloproteinases (MMPs) and, in particular, the membrane-bound MT1-MMP (MMP-14) collagenase. Inducible and selective ablation of infiltrating MoMFs negated the increased “on-fiber” accumulation of MMP-14-expressing MoMFs and the accelerated collagenolytic activity observed in the anti-LOXL2-treated mice. Many studies of liver fibrosis focus on preventing the progression of the fibrotic process. In contrast, the therapeutic mechanism of LOXL2 inhibition presented herein aims at reversing existing fibrosis and facilitating endogenous liver regeneration by paving the way for collagenolytic macrophages.

## Introduction

Liver fibrosis is a dynamic process characterized by increased deposition of extracellular matrix (ECM). It emanates from chronic liver injury of any etiology, including chronic viral infection, alcoholic liver disease (ALD), and non-alcoholic steatohepatitis (NASH), a progressive form of fatty liver disease. The liver is primed to respond quickly to injury by activating regenerative feed-forward mechanisms after eliminating the cause of injury. Yet, in the case of liver fibrosis, persistent injury triggers a chronic wound-healing response, leading to the replacement of parenchymal cells by ECM components. Progressive ECM accumulation gradually generates cirrhosis, characterized by disruption of the hepatic architecture and subsequent altered blood flow leading to portal hypertension. Decompensated liver fibrosis may cause further medical complications including ascites, hepatic encephalopathy, variceal hemorrhage, and an increased individual risk of hepatocellular carcinoma (HCC) (1). While advanced liver fibrosis and cirrhosis have been considered static and irreversible stages, the current paradigm argues that these processes are dynamic and potentially reversible that can be modulated by halting their progression and/or by promoting their resolution (2).

Collagen crosslinking is a hallmark phenotype and an essential process in fibrotic matrix stabilization, contributing to fibrosis progression and limiting its reversibility (3). During fibrosis, activated hepatic stellate cells (HSCs) transdifferentiate into proliferative, contractile, fibrogenic myofibroblasts (4). Together with portal fibroblasts, HSCs secrete large amounts of ECM proteins, predominantly fibrillar type I and III collagens (4, 5), as well as enzymes that stabilize these ECM components via crosslinking (6). Enzymes belonging to the lysyl-oxidase (LOX) family are responsible for collagen as well as elastin crosslinking in pathological conditions like fibrosis (3, 7). Particularly in the liver, crosslinking and overexpression of tissue inhibitors of metalloproteinases (TIMPs) confer resistance to proteolytic degradation, thereby promoting excess ECM accumulation and stability. Among the five variants of the LOX family, lysyl oxidase-like 2 (LOXL2) has been identified as the primary enzyme promoting network formation of collagen and elastin fibers during human and experimental liver fibrosis of various etiologies (3, 6, 8–10) as well as HCC metastasis (11). Indeed, previous studies in rodent models have indicated that LOXL2 inhibition can ameliorate liver fibrosis (9, 12), thus highlighting its therapeutic potential.

Hepatic macrophages are a heterogeneous cell population of resident self-sustaining phagocytes termed Kupffer cells (KCs) and monocyte-derived macrophages (MoMFs) recruited from the circulation to the injured liver (13, 14). Macrophages were shown to play distinct and opposing roles during liver fibrosis, having been critically implicated in both pro-fibrogenic processes and scar-tissue degradation. On the one hand, they promote fibrosis by secreting pro-fibrotic mediators such as transforming growth factor beta (TGF-β) and platelet-derived growth factor (PDGF), and indeed the targeted deletion of liver-infiltrating Ly6C^hi^ monocytes or inhibition of their recruitment ameliorates hepatic inflammation and fibrosis (15–20). On the other hand, it has been shown in mouse models that macrophages can undergo a phenotypic switch during the disease process of liver fibrosis. If chronic injury ceases, local molecular signals trigger the transition of pro-fibrogenic Ly6C^hi^ monocytes into pro-restorative Ly6C^lo^ MoMFs. These cells facilitate the resolution of fibrosis by producing specific matrix metalloproteinases (MMPs) and other proteolytic enzymes like cathepsins and are capable of both degrading and clearing fibrotic ECM (18, 19, 21–24). Indeed, depletion of MoMFs during the resolution phase exacerbates fibrosis (16, 18) while their augmentation accelerates its resolution (22, 25). Accordingly, novel strategies to treat liver disease aimed at targeting macrophages were proposed (26). Yet, with the progression of liver fibrosis, macrophages fail to engage in reparative activities. It has been shown *in vitro* that crosslinking in collagenous scaffolds limits their degradation by macrophages (27). Therefore, LOXL2-driven collagen crosslinking during liver fibrosis may impede the collagenase activity of MoMFs and their reparative behavior.

Here, we used a novel anti-LOXL2 monoclonal antibody, GS341, targeting the catalytic site of extracellular LOXL2 enzymes within the tissue. Its administration following the induction of carbon tetrachloride (CCl_4_)-induced liver fibrosis was sufficient to accelerate liver resolution by degrading scar tissue. We show that inhibition of LOXL2-mediated collagen crosslinking facilitates the arrival of MoMFs expressing a unique repertoire of collagenolytic MMPs to the proximity of collagen fibers.

## Materials and Methods

### Animals

The following 8 to 12-week-old mouse strains were used: C57BL/6J wild-type male mice were purchased from Envigo Laboratories (Jerusalem, Israel); *Cx*_3_*cr1*^*gfp*/+^ male mice (B6.129P- Cx3cr1tm1Litt/J) (28) were generously provided by Prof. Steffen Jung (Weizmann Institute of Science, Israel). All experiments and procedures were approved by the Weizmann Institute of Science Animal Care and Use Committee (IACUC approval no. 33070117-2).

### Liver Injury

Hepatic fibrosis was induced by intraperitoneal (i.p.) injections of CCl_4_ (Sigma-Aldrich, Rehovot, Israel) diluted in olive oil (Sigma-Aldrich, Rehovot, Israel) (0.9 CCl_4_ μl/g), twice a week for 4 weeks (nine injections in total).

### Therapeutic Anti-lysyl Oxidase Like 2 or Immunoglobulin G Control Antibody Treatment

In-house designed and generated antibodies (Abs) anti-LOXL2 (GS341) and anti-glutathione S-transferase [GST, immunoglobulin G (IgG) control antibody] were purified as previously described (29), both in the same conditions. Two weeks after CCl_4_-induced fibrosis, mice were injected with GS341 or control Ab every other day at a concentration of 10 mg/kg body weight, so that the last injection was given 24 h after the last injection of CCl_4_ (eight injections in total).

### MC-21 Administration

Mice received an i.p. injection of 300 μl anti-mouse CCR2 mAb (clone MC-21)-conditioned media (29 μg Ab/ml) for four consecutive days before harvesting the liver tissues.

### *In situ* Zymography

*In situ* zymography was conducted as previously described (30). Briefly, unfixed 10 μm frozen mouse liver sections were incubated with diluted DQ collagen type I (Invitrogen) (diluted 1/50 in developing buffer: 150 mM NaCl, 5 mM CaCl_2_, 100 mM Tris-HCl pH 7.6, 20 μM ZnCl, 0.05% Brij 35) for 4 h at 37°C. Next, sections were fixed with 4% paraformaldehyde, then mounting solution (Immu-Mount^TM^ Thermo Scientific) was added, and slides were covered with a coverslip. The slides were imaged under a two-photon microscope (2PM:Zeiss LSM 510 META NLO) or a Nikon Eclipse 8O-I fluorescence microscope equipped with a Nikon digital camera (DXM1200F).

### Two-Photon Microscopy, Second Harmonic Generation Imaging

Stained liver sections were imaged using a two-photon microscope in a second harmonic generation (SHG) mode: 1. 2PM:Zeiss LSM 510 META NLO, equipped with a broadband Mai Tai-HP-femtosecond single box tunable Ti-sapphire oscillator with automated broadband wavelength tuning 700–1,020 nm from Spectraphysics, for two-photon excitation. 2. Leica TCS SP8 MP in an upright configuration, equipped with a Chameleon Vision II femtosecond tunable laser (680–1,080 nm) (Coherent Inc., USA) and an Acusto Optical Tunable Filter (Leica Microsystems CMS GmbH, Germany). For second-harmonic imaging of collagen, a wavelength of 800–855 nm was used (detection at 390–450 nm).

### Calculating Co-localization of Zymography and Collagen Signals

Images of collagen fibers and zymography signals were obtained using a two-photon 2PM:Zeiss LSM 510 META NLO microscope. Collagen fibers were detected by second-harmonic imaging with a wavelength of 800–855 nm and detection at 390–450 nm. The zymography signal was excited at 488 nm, and its emission was detected at 515 nm. Analysis of the images was done by measuring the intensity of the zymography signal overlapping with the main collagen fiber in the image. Analysis was done with ImageJ software.

### Cell Line and Culture

The human dermal fibroblast (HDF) cell line was a gift from the laboratory of Stephen Weiss (University of Michigan, Ann Arbor, MI). HDF cells were cultured in high-glucose Dulbecco's modified Eagle's medium (DMEM, Invitrogen) supplemented with 10% (v/v) heat-inactivated fetal bovine serum (FBS, Invitrogen), 100 U/ml penicillin, and 100 g/ml streptomycin (Biological Industries). The cells were maintained at 37°C in a humidified atmosphere containing 5% CO_2_, and the medium was exchanged every 2–3 days and passaged after reaching 80–90% confluence. For ECM synthesis, HDF cells were grown on glass coverslips in 24-well dishes until reaching contact inhibition. Then, the medium was replaced and supplemented with 5 ng/ml epidermal growth factor (EGF), 5 μg/ml insulin, and 100 μg/ml l-ascorbic acid phosphate magnesium salt n-hydrate to induce ECM secretion, in the presence of phosphate buffered saline (PBS) or GS341 in PBS (100 ng/μl) for 14 days.

### Immunoprecipitation

Magnetic protein G beads (Genescript) were incubated with GS341 according to the manufacturer's instructions. The GS341-coated beads were incubated with a fibrotic 48 h liver tissue lysate for 1 h at room temperature. Pellet beads were obtained by magnet separation rack and then were washed three times with PBS. The immunoprecipitation complex was eluted by adding 90 μl of elution buffer (Thermo-Scientific) directly to the beads followed by 5-min incubation. pH neutralization was performed by adding 10 μl of 1 M Tris-HCl pH 8.

### Histopathological Fibrosis Scoring and Calculation of Collagen Coverage Area

Liver samples were fixed with 4% paraformaldehyde, paraffin embedded, sectioned, and stained with Sirius red. Sirius red-covered areas were analyzed and quantified by ImageJ software: for all images, color de-convolution of FastRed FastBlue 3,3′-diaminobenzidine (DAB) was applied. Then, for each red image, a suitable threshold was applied, and Analyze Particles plugin was used to detect the collagen-covered area. Blind grading of liver fibrosis severity was performed by a trained pathologist based on the Ishak histopathological scoring method (31). In brief, fibrosis was scored as 0 (no fibrosis), 1 (some portal tracts expanded), 2 (most portal tracts expanded), 3 (most portal tracts expanded, ± links), 4 (marked bridging, P-P and P-C links), 5 (marked bridging, occasional nodules, incomplete cirrhosis), and 6 (cirrhosis, probable or definite).

### Quantitative Real-Time PCR

Liver tissues were homogenized using a bead beater homogenizer. Total RNA was isolated using the PerfectPure RNA Tissue Kit (5 Prime GmbH) according to the manufacturer's protocol. RNA was reverse transcribed using High-Capacity cDNA Reverse Transcription Kit (Applied Biosystems Inc.). qRT-PCR was performed using SYBR Green PCR Master Mix (Applied Biosystems inc.) on an ABI 7300 instrument (Applied Biosystems). Values were normalized to the *Tbp* or *Rplp0* housekeeping genes. Primer sequences are listed in Table 1 below. Data are presented as mean fold change using the 2-Δ^CT^ method (32). The standard error of the mean (SEM) was calculated on the 2^−ΔCT^ data, as was the statistical analysis.

**Table 1:** Primer sequences used for qRT-PCR.

**Gene**	**Forward**	**Reverse**
Mouse *tbp*	5′-GAAGCTGCGGTAC AATTCCAG-3′	5′-CCCCTTGTACCCT TCACCAAT-3′
Mouse *rplp0*	5′-TCCAGCAGGTGTT TGACAAC-3′	5′-CCATCTGCAGACA CACACT-3′
Mouse *acta2*	5′-GTCCCAGACATCAG GGAGTAA-3′	5′-TCGGATACTTCAGC GTCAGGA-3′
Mouse *col1a1*	5′- GCTCCTCTTAGG GGCCACT-3′	5′-CCACGTCTCACC ATTGGGG-3′
Mouse *timp1*	5′-CGAGACCACCTTA TACCAGCG-3′	5′-ATGACTGGGTGTA GGCGTA-3′

### De-cellularization of Liver Tissues

Samples were incubated in a de-cell solution with 3% Triton-100 (6 h, 25°C) and then in a de-cell solution with 0.4% Triton-100 overnight at 4°C [de-cell solution: 1.5 M NaCl, 50 mM Tris pH 8, 50 mM EDTA, protease inhibitor cocktail (Roche)]. Samples were washed three times in ddH_2_O and then incubated with 0.5% sodium deoxycholate (60 min, 25°C) to remove lipid remaining. Samples were washed again three times in ddH_2_O and stored at 4°C until use.

### Scanning Electron Microscope

De-cellularized mouse liver tissues were fixed using fixative buffer (4% paraformaldehyde, 2% glutaraldehyde in 0.1 M cocodylate buffer with 5 mM CaCl_2_ pH 7.4) overnight at 4°C. The fixed samples were incubated with 1% uranyl acetate for 30 min in the dark and then dehydrated through increasing concentrations of ethanol ranging from 30 to 100%. Samples were subsequently dried in a critical point dryer and gold sputtered for imaging by a scanning electron microsope (SEM) (Ultra 55 Feg; ZEISS). Fibrotic fiber thickness from high-resolution SEM images was measured. For each image, fibers were randomly chosen and measured using ImageJ software. As this is a continuous model of fibrosis, we assume new fibers are always being formed; therefore, fibers under thickness of 25 μm were omitted from total calculation.

### Cell Isolation for Flow Cytometry and Mass Cytometry

Mice were sacrificed, and their livers were harvested after perfusion with PBS via the left ventricle. Livers were weighed, minced into small fragments, and incubated in shaking for 45 min at 37°C with 1 ml PBS (with Mg^++^ and Ca^++^) containing 0.5 mg/ml collagenase type IV (Sigma-Aldrich, Rehovot, Israel) and 0.1 mg/ml DNase I (Roche). Digested tissue was filtered and mashed with a syringe plunger through a 250-μM nylon sieve in FACS buffer [PBS, 2% fetal calf serum (FCS), 2 mM ethylenediaminetetraacetic acid (EDTA)] to mechanically dissociate the remaining tissue. This was followed by three cycles of washing with FACS buffer at 30 g, each time taking only the supernatant, while omitting the non-leukocyte cell pellet. The supernatant cell pellet was then centrifuged at 390 g, and the pellet cells were lysed for erythrocytes using a red blood cell lysis buffer (Sigma-Aldrich, Rehovot, Israel) (2 min, 25°C).

### Flow Cytometry

The following anti-mouse Abs were used: CD45 (clone 30-F11), CD11b (clone M1/70), Ly6C (clone HK1.4), Ly6G (clone 1A8), CX_3_CR1 (clone SA011F11), CD64 (clone X54-5/7.1), MHCII (clone M4-114.15.2)—all purchased from BioLegend (San Diego, USA). Anti-mouse F4/80 (clone A3-1) was purchased from BIORAD. Anti MMP-14 was purified in-house from hybridoma cells of LEM-2/15 (33). The cells were incubated with Abs for 30 min in FACS buffer (dark, 4°C) and then washed once with FACS buffer. Cells were analyzed with BD FACSCanto™ II (BD Bioscience). Flow cytometry analysis was performed using FlowJo software (TreeStar, Ashland, OR, USA).

### Mass Cytometry (cyTOF)

Subsequent to the cell isolation procedure, cells were stained according to a previously published protocol (34). Individual mice cell suspensions were stained with 0.125 μM Cell-ID Cisplatin for viability and fixed using Maxpar® Fix I Buffer. Samples were then permeabilized using Maxpar® Barcode Perm Buffer and then barcoded using the Cell-ID™ 20-Plex Pd Barcoding Kit, allowing us to join samples for antigen staining. Abs used for staining are listed in Table 2 below. Before analyzing, the cell suspension was incubated with Cell-ID Intercalator Iridium for 20 min. Cells were analyzed with a cyTOF2® mass cytometer (Fluidigm). Results were normalized and debarcoded using fluidigm cyTOF software (35). Gating and further analysis of the CyTOF results were done with FlowJo software.

**Table 2:** List of antibodies used in the mass cytometry analysis.

**Name**	**Product**	**Provider**	**Isotope conjugation**
Anti-human/mouse CD45R/B220	3160012	FLUIDIGM	160Gd–FLUIDIGM
Anti-mouse CD11c	3142003	FLUIDIGM	142Nd–FLUIDIGM
Anti-mouse TER-119	3154005	FLUIDIGM	154Sm–FLUIDIGM
Anti-mouse Ly-6C	3162014	FLUIDIGM	150Nd–FLUIDIGM
Anti-mouse Ly-6G	3141008B	FLUIDIGM	141Pr–FLUIDIGM
Anti-mouse CD64	3151012	FLUIDIGM	151Eu–FLUIDIGM
Anti- mouse F4/80 (BM8)	3159009	FLUIDIGM	159Tb–FLUIDIGM
Anti-mouse CD45	3089005	FLUIDIGM	89Y–FLUIDIGM
Anti-mouse CD3e (maxpar ready)	BLG-100345	Biolegend	153Eu–FLUIDIGM
Anti-mouse CX3CR1 (SA011F11)	3164023	FLUIDIGM	164Dy–FLUIDIGM
Anti-mouse I-A/I-E (M5/114.15.2)	3209006B	FLUIDIGM	209Bi–FLUIDIGM
Anti-mouse CD11b	3143015	FLUIDIGM	143Nd–FLUIDIGM
Anti-MMP-9	ab38898	Abcam	149Sm–Home made
Anti-MMP-14	ab51074	Abcam	156Gd–Home made

### Immunofluorescence Staining

#### Frozen Section

Liver samples embedded in OCT were cross-sectioned (10 μm) on glass microscope slides. Sections were fixed with PBS 4% paraformaldehyde (PFA) for 20 min at 25°C. Samples were blocked in PBS, 20% normal horse serum, and 0.2% Triton x-100 (20 min, 25°C) and then incubated with a primary Ab in PBS, 2% normal horse serum, and 0.2% triton (overnight, 4°C). Samples were then washed three times in PBS and incubated with a secondary antibody (60 min, 25°C). Next, they were mounted in a mounting medium.

#### Paraffin-Embedded Sections

Liver samples were fixed with PBS 4% PFA, paraffin embedded and sectioned (4 μM). Slides were de-paraffinized using Wcap solution (Bio optica, Milano, Italy) (75°C, 20 min). Antigen retrieval was performed in 0.1 M EDTA, pH 8.0 (Diagnostic BioSystems, CA, USA) using a pressure cooker (125°C, 3 min), followed by washes with worm ddH_2_O. Samples were blocked in PBS, 20% normal horse serum, and 0.2% Triton X-100 (20 min, 25°C) and then incubated with primary Ab in PBS, containing 2% normal horse serum and 0.2% Triton X-100 (60 min, 25°C). Next, samples were washed three times in PBS and incubated with a secondary antibody (60 min, 25°C) and mounted in a mounting medium. Primary Abs: anti-GFP antibody (ab6673, Abcam), anti MMP-14 (ab51074, Abcam), TIMP1 (ab86482, Abcam). For MMP-14 staining, the samples were incubated with a biotin antibody (711-065-152, Jackson ImmunoResearch) following the incubation of the first Ab. Then, a cy3-streptavidin (016-160-084, Jackson ImmunoResearch) was used as a secondary Ab. TIMP1 staining was done following *in situ* zymography after the sections were fixed with 4% PFA.

### Tissue Extraction and Western Blotting

Frozen liver tissues were washed in PBS, homogenized in RIPA lysis buffer (EMD Millipore, Burlington, MA, USA) with a protease inhibitor (Roche, Basel, Switzerland) using a hand homogenizer, and centrifuged (14,000 *g*, 15 min, 4°C). Supernatants were then resuspended in a sample buffer [200 mM Tris pH 6.8, 40% glycerol, 8% sodium dodecyl sulfate (SDS), 100 mM dithiothreitol (DTT), 0.2% bromophenol blue] and boiled for 5 min. Tissue extracts were then subjected to SDS-polyacrylamide gel electrophoresis (PAGE) and transferred onto polyvinylidene fluoride (PVDF) membranes by electro-blotting. Membranes were blocked in Tris-buffered saline with Tween 20 (TBST) buffer (200 mM Tris pH 7.5, 1.5 M NaCl, 0.5% Tween 20) and 2% bovine serum albumin (BSA, 60 min, 25°C) and then incubated with the corresponding primary Ab (60 min, 25°C), washed three times with TBST and incubated with horseradish peroxidase (HRP)-conjugated secondary antibody (60 min, 25°C). Quantification of the band intensities was performed using the ImageJ analysis tool. Abs used in this study: LOXL2 (ab179810, Abcam), LOX (ab ab174316, Abcam), and glyceraldehyde 3-phosphate dehydrogenase (GAPDH, sc-25778, Santa Cruz). Secondary Abs (both anti-rabbit and mouse) conjugated to HRP were purchased from Jackson ImmunoResearch (cat No.111-001-003 and 115-001-003, respectively). Abs were used at the manufacturer's recommended dilution.

### Analysis of Macrophages/MMP-14 Distance From Collagen Fibers

Z-stack images of liver sections were obtained with a Leica TCS SP8 MP microscope. Collagen fibers were detected by second harmonic imaging with a wavelength of 855 nm and detection at 390–450 nm. The macrophage signal [green fluorescent protein (GFP)] was excited at 488 nm, and its emission was detected at 515 nm. The MMP-14 signal (cy3) was excited at 561 nm, and its emission was detected at 570–590 nm. Analysis of the images was done as follows: in Imaris software, surfaces were created for the main fiber in the image (omitting the surrounding short fibrils) and for the macrophages/MMP-14, both with a suitable threshold. A dilate surface of 15 μm and distance transformation map around the main fiber surface were created in order to analyze the distance of the macrophages/MMP-14 from the main fiber. Finally, the total macrophages/MMP-14 volume at a distance of ≤ 15 μm from fiber was normalized to the volume of the fiber 15 μm dilate surface.

### Statistical Analysis

Data were analyzed by unpaired, two-tailed *t*-test to compare between two groups or by one-way ANOVA to compare several groups. After the null hypothesis was rejected (*p* < 0.05), Tukey's Honestly Significant Difference or Dunnett tests were used for follow-up pairwise comparison of groups in the one-way ANOVA. Data are presented as mean ± SEM; values of *p* < 0.05 were considered statistically significant (^*^P < 0.05, ^**^P < 0.01, ^***^P < 0.001).

## Results

### Collagenolytic Activity of Matrix metalloproteinases During the Recovery Phase of Carbon Tetrachloride-Induced Liver Fibrosis

**Figure 1:**
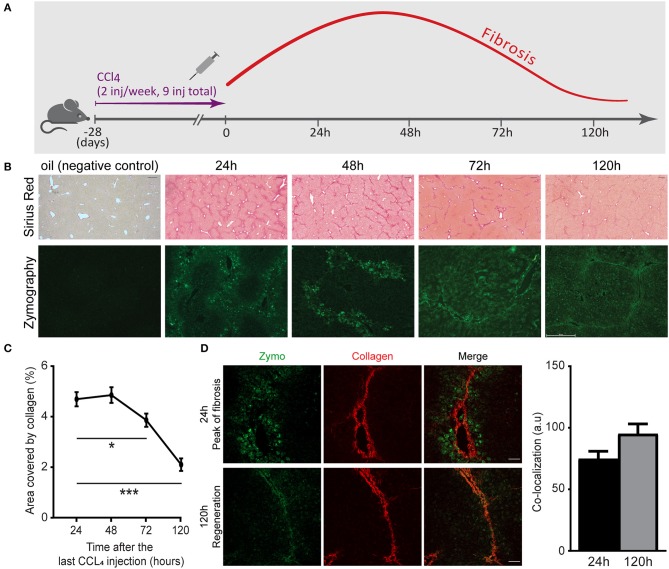
Matrix metalloproteinase (MMP)-governed collagenolytic activity naturally appears at the resolution phase of carbon tetrachloride (CCl_4_)-induced fibrosis. **(A)** Schematic representation of the liver fibrosis model. inj, injection. **(B)** Representative images of Sirius red stain (top) and *in situ* zymography (bottom) of samples collected from mice sacrificed at different time points after the last (ninth) injection of CCl_4_ or oil control (Sirius red scale−250 μm; zymography scale−50 μm). **(C)** Sirius red quantification by calculating the fraction of collagen-covered area. Analysis was done using ImageJ software (*n* = 4). **(D)** Representative images of livers excised from CCl_4_-injected mice at the peak of fibrosis (24 h) and recovery phase (120 h). Zymography signal (green) and collagen signal by second harmonic generation (SHG, red) were obtained using two-photon microscopy (scale−50 μm) (*n* ≥ 2). The co-localization of the zymography and collagen signals were quantified by measuring the intensity of the zymography signal that overlapped with the main collagen fiber in the image. Data were analyzed by one-way ANOVA with Dunnett *post hoc* comparison. Results are presented as mean ± SEM with significance: *p < 0.05, ***p < 0.001. Data in **(A–D)** represent a single experiment.

Understanding the naturally occurring collagenolytic events in the fibrotic liver necessitates a reproducible model of reversible hepatic fibrosis. Hence, we have utilized a spontaneously reversible murine model of liver fibrosis based on repeated challenges with CCl_4_. C57BL/6 mice received nine i.p. injections of CCl_4_ over the course of 4 weeks. Liver tissue was then harvested 24, 48, 72, and 120 h after the last injection (Figure 1A). Comparison was made with age-matched uninjured livers from mice injected with vehicle (oil) only. Analysis of Sirius red staining, which stains for fibrillary collagen, revealed fibrosis peaks ~24 h after the last injection, manifested by bridging fibrosis (collagen fibers connecting at least two portal triads) and septa formation (Figures 1B,C). Early regeneration took place after 72 h and at 120 h, when a substantial reduction in fibrosis (area covered by collagen) had already occurred (Figures 1B,C). Several groups have shown that a myriad of ECM remodeling enzymes are elevated during liver fibrosis, including ECM-degrading enzymes such as MMPs. However, their contribution to the pathogenesis of liver fibrosis or its resolution remains controversial (36, 37). We therefore investigated the localization and kinetics of collagenolytic MMPs following the cessation of CCl_4_ administration by testing their *in situ* activity. To this end, non-fixed liver samples were treated with a fluorogenic collagen substrate that emits a fluorescent signal upon its *in situ* digestion by endogenous MMPs. At the peak of fibrosis (24 and 48 h), collagenolytic enzyme activity was demonstrated only at the area surrounding the collagen fibers. However, at 72–120 h, the collagenolytic activity of MMPs was associated with the collagen fibrils as depicted by the co-localization of the zymographic signal with the second harmonic generation (SHG) signal (Figures 1B,D). These results highlight that access of natural collagen-degrading enzymes to the fibrotic scar commences at 72–120 h following termination of CCl_4_ treatment.

### GS341 Relaxes Collagen Packaging *in vitro* and Specifically Targets Lysyl Oxidase Like 2 in the Fibrotic Liver

**Figure 2:**
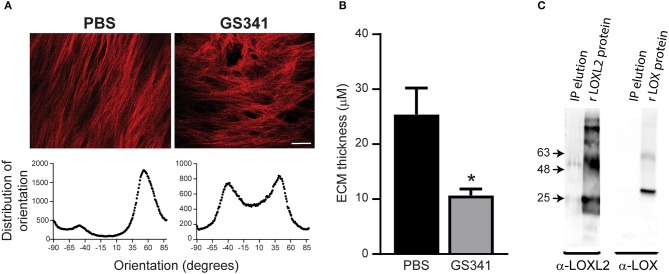
GS341 antibody targets specifically lysyl oxidase like 2 (LOXL2) and affects collagen morphology. **(A)** Upper panel: Representative second harmonic generation (SHG) images of an *in vitro* native fibroblast-derived three-dimensional (3D) matrix treated with GS341 or phosphate buffered saline (PBS) (scale−50 μm). Lower panel: Representative fiber directionality analysis plots depicting the frequency of fibers in a specific orientation. Analysis was done using ImageJ software. **(B)** The thickness of the *in vitro* native fibroblast-derived 3D matrix treated with GS341 or PBS. **(C)** Immunoprecipitation (IP) of fibrotic liver lysate with GS341. Beads conjugated with GS341 were incubated with a fibrotic liver lysate, and the eluted fraction was tested in Western blot against commercial anti-LOX and LOXL2 antibodies (lane 1 in each membrane). LOX and LOXL2 recombinant proteins (lane 2) served as positive controls. IP, immunoprecipitation; r, recombinant. Data were analyzed via an unpaired, two-tailed *t*-test. Results are presented as mean ± SEM with significance: *p < 0.05. Data in **(A–C)** represent one single experiment.

Targeting specific stages of collagen assembly during liver fibrosis presents a great challenge due to the involvement of various crosslinking enzymes in the multistep, hierarchical process of collagenous matrix buildup. LOXL2 plays a critical role in collagen crosslinking during liver fibrosis development, with its inhibition linked with reduced liver fibrosis (9, 12). Here, we used a novel anti-LOXL2 monoclonal antibody (mAb) developed in our lab, GS341, which targets the catalytic site of the enzyme (29). Grossman et al. (29) have previously shown that GS341 changes the natural alignment and diameter of collagen fibers *in vitro* as well as *in vivo* in a breast cancer model. In agreement with these previous results (29), we found that LOXL2 inhibition by GS341 in a native fibroblast-derived three-dimensional (3D) matrix *in vitro* system alters the natural alignment of fibrillary collagen (Figure 2A). In addition, ECM scaffolds treated with GS341 were significantly thinner than the control (Figure 2B). Next, in order to verify the specificity of the GS341 mAb within the fibrotic liver, immunoprecipitation (IP) was conducted (Figure 2C). GS341 specifically precipitated LOXL2 from the lysate and not LOX (Figure 2C). Altogether, these results display the specificity of GS341 toward LOXL2 in the fibrotic liver and its ability to interfere with collagen alignment and packaging.

### Inhibition of Lysyl Oxidase Like 2-Mediated Collagenous Matrix Remodeling Ameliorates Hepatic Fibrosis

**Figure 3:**
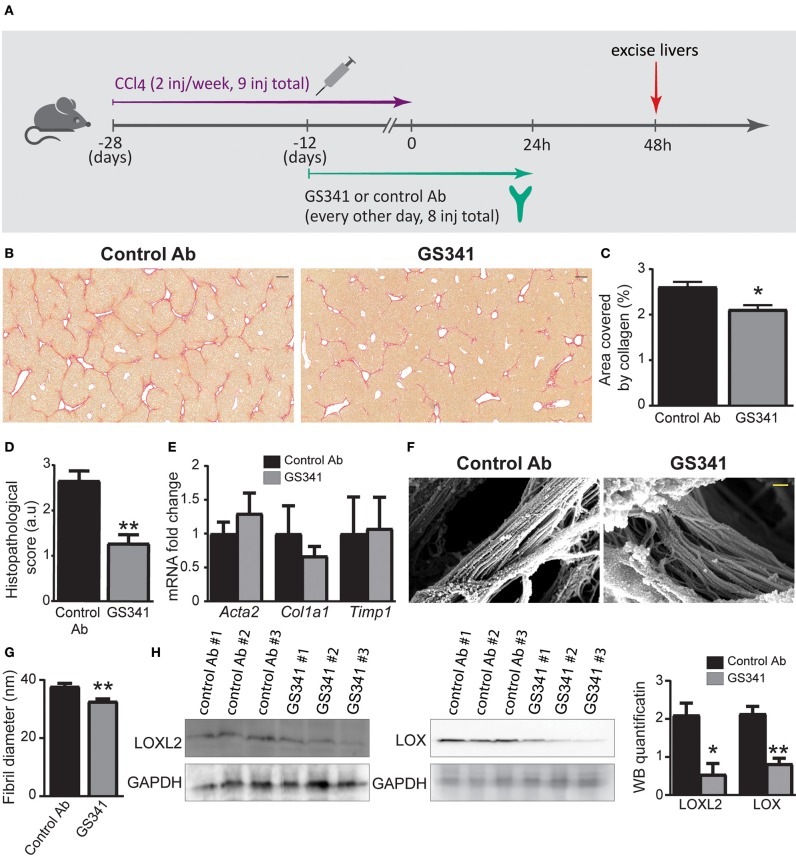
Inhibition by GS341 demonstrates improved recovery in chronic carbon tetrachloride (CCl_4_)-induced liver fibrosis and morphological change to fibrotic fibers. **(A)** Schematic representation of the experimental setting. inj, injection. **(B)** Representative images of Sirius red staining performed on paraffin-embedded slides of livers from GS341-treated and control Ab-treated mice. Livers were excised at 48 h after the last (ninth) injection of CCl_4_ (scale−200 μm). **(C)** Quantification of the percentage area covered by collagen, performed by ImageJ. **(D)** Graph showing histopathological scoring in GS341-treated mice compared to control Ab-treated mice (**C,D**–*n* ≥ 6). **(E)** qRT-PCR analysis of fibrosis marker genes at 24 h after CCl_4_-induced liver fibrosis (*n* = 6). **(F)** Representative SEM images of de-cellularized three-dimensional (3D) extracellular matrix (ECM) liver scaffolds excised 48 h after the last CCl_4_ injection (scale−200 nm). **(G)** Quantification of fiber thickness in the scanning electron microscope (SEM) images was performed using ImageJ software. Comparison of fibril diameters in control vs. GS341-treated samples shows significant reduction of the fibril diameters of the latter. Statistics are obtained from the four biologic replicates for each case, 12 images per treatment (*n* = 4). **(H)** Western blotting (WB) with commercial anti-lysyl oxidase like 2 (LOXL2) and anti-LOX antibodies (Abs) of liver samples treated with control Ab or GS341, excised 48 h after the last CCl_4_ injection. LOXL2/LOX expression was normalized to glyceraldehyde 3-phosphate dehydrogenase (GAPDH) expression. Quantification of the WB was done using ImageJ software. Data were analyzed by unpaired, two-tailed *t*-test. Results are presented as mean ± SEM with significance: *p < 0.05, **p < 0.01. Data in **(A–D)** represent three independent experiments. Data in **(E–G)** represent two independent experiments. Data in **(H)** represent one single experiment.

We further investigated the ability of GS341 to ameliorate existing progressive fibrosis. Accordingly, mice on the CCl_4_ regimen started after 2 weeks to receive either GS341 or an IgG control mAb every other day (eight injections in total), with the last treatment given 24 h after the last CCl_4_ injection (Figure 3A). Analyses were performed at 48 h after the last CCl_4_ injection. This time point is at the peak of the inflammatory/fibrotic phase, while resolution and “on fiber” collagen degradation activity has not yet begun (Figures 1B–D). Sirius red staining revealed a significant reduction in the collagen-stained area in mice following GS341 compared to control Ab treatment (Figures 3B,C; Figure S1A). Blind grading of liver fibrosis severity performed by a trained pathologist based on the Ishak histopathological scoring method (31) showed that mice treated with GS341 exhibited a significantly improved fibrotic score mainly due to reduced bridging scar tissue between portal tracts (Figure 3D). We have repeated this experiment, this time looking at 24 h following the last CCL_4_ injection, to examine the cumulative effect of GS341 on collagen accumulation. Sirius red staining revealed a significant reduction in collagen-stained area in the GS341 group (Figures S1B,C). Interestingly, qRT-PCR of liver tissue 24 h post treatment revealed no difference between the GS341-treated and control groups in the gene expression of key fibrotic elements, including collagen type-I (*Col1a1*), alpha-SMA (*Acta2*), and tissue inhibitor of metalloproteinases 1 (*Timp1*) (Figure 3E), suggesting that LOXL2 inhibition in this model and under this treatment regimen does not directly affect the fibrotic activity of HSCs. Following up on the observation that LOXL2 inhibition affected collagen morphology *in vitro* (Figures 2A,B), we assessed its effect on collagen arrangement and assembly *in vivo*. High-resolution SEM analysis of de-cellularized 3D ECM scaffolds extracted from GS341- and control Ab-treated livers 48 h after the last CCl_4_ injection uncovered major changes in the morphology and arrangement of collagen fibers (Figure 3F). While collagen fibers in the control livers displayed a tightly packed structure of linear fibrils, those in the GS341-treated livers assumed a looser, disoriented structure with gaps between the fibrils (Figure 3F). A significant reduction in average collagen fibril diameter was observed in control (37 nm) vs. GS341 (33 nm)-treated groups (Figure 3G). Interestingly, reduced levels of both LOXL2 and LOX were observed in the GS341-treated livers, indicating that inhibition of extracellular LOXL2 downregulates both collagen cross-linkers (Figure 3H). Overall, these results demonstrate that inhibition of LOXL2 using our novel active-site neutralizing mAb reduces the amount of fibrotic collagenous matrix in liver fibrosis and modifies its arrangement and assembly.

### Lysyl Oxidase Like 2 Inhibition Turns Collagen Fibers More Accessible to Macrophages

**Figure 4:**
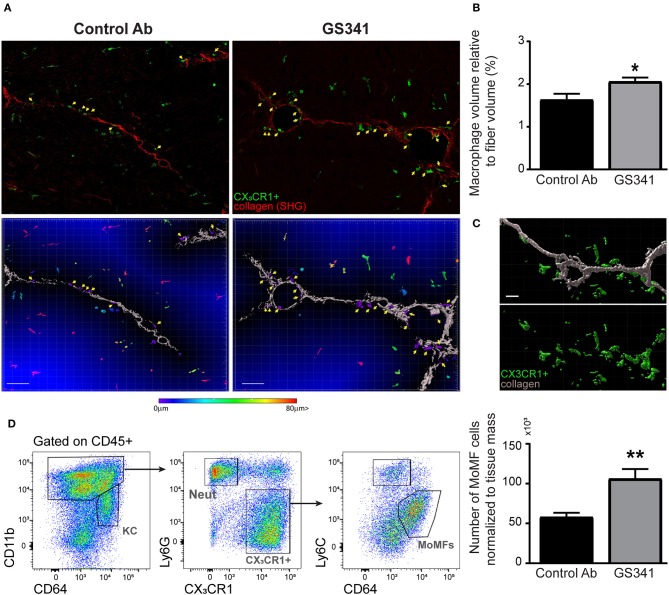
Inhibition of lysyl oxidase like 2 (LOXL2)-governed extracellular matrix (ECM) crosslinking paves the way for macrophages to the fibrotic fibers. *CX*_3_*CR1*^*gfp*/+^ transgenic reporter mice subjected to carbon tetrachloride (CCl_4_)-induced liver fibrosis and, after 2 weeks, to treatment with GS341 or a control antibody (Ab). Livers were excised 48 h following the last CCl_4_ injection, stained with anti-green fluorescent protein (GFP) antibody, and then two-photon microscopy imaging was used for the mutual detection of CX_3_CR1-GFP expression and the second harmonic generation (SHG) signal at the portal tract fibrotic area. **(A)** Upper panel: representative images of the distribution of CX_3_CR1-GFP^+^ monocyte-derived macrophages (MoMFs) (green) near collagen fibers (red) in GS341- and control Ab-treated mice; yellow arrows indicate CX_3_CR1-GFP^+^ MoMFs adjacent to the fibers; scale−50 μm. Lower panel**:** representative images of Imaris analysis for quantification of the distance of infiltrating CX_3_CR1-GFP^+^ MoMFs from the fiber. CX_3_CR1-GFP^+^ MoMFs are color coded according to their distance from the fiber; yellow arrows indicate CX_3_CR1-GFP^+^ macrophages adjacent to the fibers; scale−50 μm. **(B)** Quantification of the CX_3_CR1-GFP^+^ signal's volume within 15 μm from the fiber normalized to the total collagen fiber volume; scale−50 μm (*n* ≥ 5). **(C)** Imaris images showing that macrophages adjacent to the fiber are wrapping the fiber and embracing its shape; scale−10 μm. **(D)** Representative flow cytometry images showing the gating strategy used to identify MoMFs (CD45^+^CD11b^+^Ly6G^−^CX_3_CR1^+^Ly6C^lo^F4/80^+^CD64^+^ MHCII^+^) and a summarizing graph showing their frequency normalized to tissue mass (g) at 48 h following CCl_4_-induced liver fibrosis. KC, kupffer cells; Neut, neutrophils; MoMFs, monocyte-derived macrophages. Data were analyzed by an unpaired, two-tailed *t*-test. Results are presented as mean ± SEM with significance: *p < 0.05, **p < 0.01. Data in **(A,B)** represent a single experiment, and data in **(D)** represent three independent experiments.

The ameliorating effects of LOXL2 inhibition on the collagen coverage area, fibrotic score (Figures 3B–D), and morphology (Figures 2A,B, 3F–G) prompted us to further explore whether attenuation of LOXL2-governed crosslinking renders collagen fibers more accessible and amenable to degradation. Innate immune cells, especially macrophages, play distinct roles during liver fibrosis but bear the potential to resolve it upon termination of liver injury (16, 18, 19, 21–24). We hypothesized that LOXL2-governed crosslinking makes collagenous matrix less accessible to macrophages and their collagenolytic MMPs. Therefore, we examined the localization of macrophages in livers of *Cx*_3_*cr1*^*gfp*/+^ transgenic reporter mice (28) subjected to CCl_4_-induced liver fibrosis and treated with GS341 or a control Ab. In the liver of these mice, GFP expression can be used to trace infiltrating Ly6C^hi^ monocytes and their MoMF decedents, but not resident KCs (14, 38). Two-photon microscopy imaging was further utilized for the mutual detection of CX_3_CR1-GFP expression (mainly MoMFs and some Ly6C^hi^ monocytes at 48 h) and SHG signaling (collagen fibers) at the portal tract fibrotic area. Forty-eight hours following the last CCl_4_ injection, there was a significant increase in the representation of CX_3_CR1-GFP^+^ Ly6C^hi^ monocytes or their MoMF progenies in the vicinity of fibrotic collagen fibers in GS341-treated mice (Figure 4A). This was further calculated by the fraction of CX_3_CR1-GFP^+^ MoMF cell volume within a radius of 15 μm from the collagen fiber out of the total collagen fiber volume (Figure 4B). Interestingly, the macrophages adjacent to the fiber wrapped the fiber and embraced its shape (Figure 4C). Moreover, GS341-treated mice also exhibited increased representation of MoMFs in their livers (Figure 4D), suggesting that in the absence of LOXL2 activity, the liver is more attractive or passable for Ly6C^hi^ monocytes.

### Ly6C^hi^ Monocytes and Their Monocyte-Derived Macrophage Descendants Express a Unique Repertoire of Collagenolytic Matrix-Degrading Enzymes

**Figure 5:**
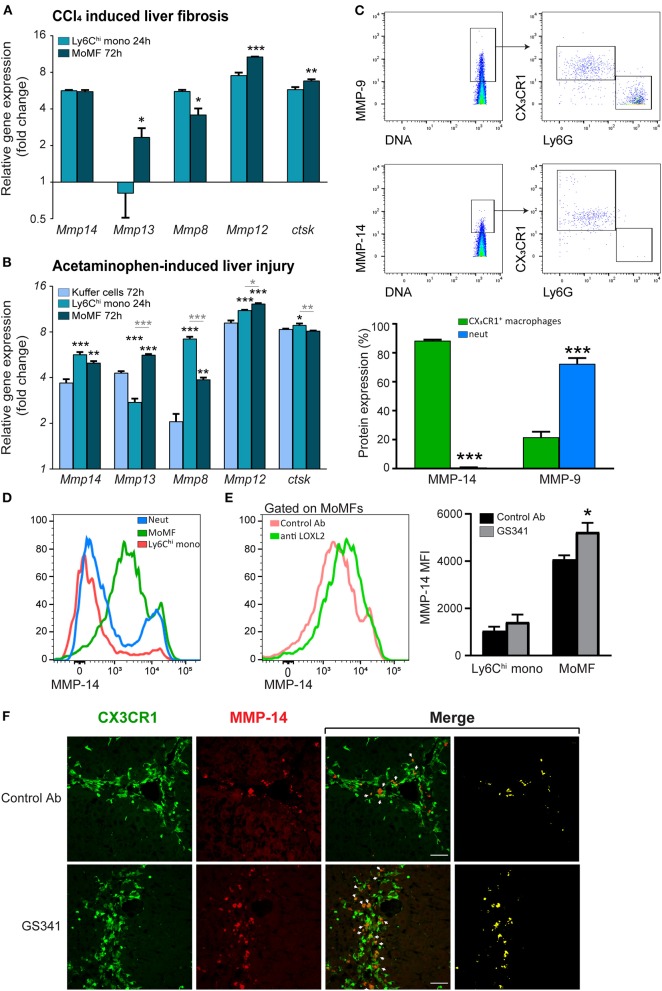
Fibrotic macrophages express different collagenolytic matrix metalloproteinases (MMPs) in injured liver. **(A)** *Mmp8, Mmp12, Mmp13, Mmp14*, and *Cstk* gene expression in sorted Ly6C^hi^ monocytes and monocyte-derived macrophages (MoMFs) 24 and 72 h following carbon tetrachloride (CCl_4_)-induced fibrosis, respectively, as extracted from the ArrayExpress database (www.ebi.ac.uk/arrayexpress, accession no. E-MEXP-3177). Their expression was normalized to the expression of the adipocyte marker *Adipoq*, which served as a background expression level (*n* = 3). **(B)** *Mmp8, Mmp12, Mmp13, Mmp14*, and *Cstk* gene expression in sorted Ly6C^hi^ monocytes 24 h following acetaminophen-induced liver injury (AILI) and in sorted Kupffer cells (KCs) and MoMFs 72 h post AILI. Data were extracted from our existing database (GSE55606). Their expression was normalized to the expression of the adipocyte marker *Adipoq*, which served as a background expression level. Black asterisks indicate a significant difference from KCs at 72 h. Gray asterisks indicate a significant difference between Ly6C^hi^ monocytes at 24 h and MoMFs at 72 h. **(C)** cyTOF analysis of livers following CCl_4_-induced liver injury. Mice were injected three times with CCl_4_ without any treatment, after which livers were excised 48 h following the last CCl_4_ injection. Presented are plots depicting the gating strategy and analysis of neutrophils and macrophages percentage from total MMP-14^+^ or MMP-9^+^ cells (*n* = 3). **(D)** Representative flow cytometry images showing the expression of MMP-14 in liver infiltrating neutrophils (CD11b^+^Ly6G^+^CX_3_CR1^−^CD64^−^), Ly6C^hi^ monocytes (CD11b^+^Ly6G^−^ CX_3_CR1^+^Ly6C^hi^CD64^lo^), and MoMFs (CD11b^+^Ly6G^−^CX_3_CR1^+^Ly6C^lo^CD64^+^) 48 h following CCl_4_-induced liver fibrosis. **(E)** Mean fluorescent intensity (MFI) expression of MMP-14 in MoMFs as depicted by flow cytometry analysis. **(F)** Representative images displaying the localization of CX3CR1-GFP^+^ MoMFs (green) and MMP-14 (red) in GS341- and control Ab-treated mice. White arrows indicate co-localization of CX3CR1-GFP^+^ and MMP-14 signals. Mono, monocyte; Neut, neutrophils; MoMFs, monocyte-derived macrophages. Data were analyzed using an unpaired, two-tailed *t*-test **(A,C,E)** or one-way ANOVA with Tukey's Honestly Significant Difference *post hoc* comparison **(B)**. Results are presented as mean ± SEM with significance: *p < 0.05, **p < 0.01, and ***p < 0.001. Data in **(A–F)** represent a single experiment.

Intact fibrillar collagen can only be cleaved by a subset of MMPs (i.e., MMP-1, MMP-8, MMP-13, and MMP-14) (37) and by other proteases, such as cathepsin K (39). Given that inhibition of LOXL2-governed buildup of collagenous matrix during liver fibrosis facilitates the arrival of CX_3_CR1^+^ Ly6C^hi^ monocytes and their MoMF progenies in the vicinity of fibrous collagen fibers (Figure 4), we investigated whether this macrophage subset expresses MMPs that can potentially degrade collagen fibers. To assess the expression of collagenolytic MMPs in these cells, we revisited available gene expression databases. In a model of reversible CCL_4_-induced liver fibrosis similar to the one used here, gene expression profiling has been done on liver infiltrating CX_3_CR1^+^ Ly6C^hi^ monocytes at the necro-inflammatory phase (24 h) and their MoMF progenies at the early resolution phase (72 h) (18). In the original paper, the expression of some collagenolytic MMPs was noted in MoMFs. Here, we revisited this database to further elaborate the full repertoire of collagenolytic matrix enzymes expressed by these cells. We found that Ly6C^hi^ monocytes upregulate the expression of MMP-13 (*Mmp13*) and downregulate that of MMP-8 (*Mmp8*) upon differentiation into pro-restorative MoMFs (Figure 5A). Both expressed pronounced levels of the membrane-type 1 (MT1-MMP) (*Mmp14*) (Figure 5A). Remarkably, MT1-MMP (MMP-14) is known to efficiently degrade fibrillary collagens *in vivo*. Similar results were obtained in a model of acetaminophen-induced liver injury (AILI) which included, in addition to Ly6C^hi^ monocytes and MoMFs, the gene expression profiling of resident KCs (14). Accordingly, gene expression profiling of liver infiltrating CX_3_CR1^+^ Ly6C^hi^ monocytes during the necro-inflammatory phase (24 h) and of resident CX_3_CR1^−^ KCs and CX_3_CR1^+^ MoMFs during the resolution phase (72 h) revealed that Ly6C^hi^ monocytes had higher levels of MMP-8 but lower levels of MMP-13 and similar levels of MMP-14. KCs expressed lower levels of all these MMPs (Figure 5B). Cathepsin K gene expression (*Ctsk*) was upregulated upon the differentiation of Ly6C^hi^ monocytes into MoMFs at the resolution phase of both CCl_4_-indcued fibrosis (Figure 5A) and AILI (Figure 5B). Elastin is another ECM protein linked to liver fibrosis progression, and macrophage-derived MMP-12 was shown to mediate its degradation during experimental liver fibrosis (23). We found that Ly6C^hi^ monocytes profoundly upregulate MMP-12 gene expression (*Mmp12*) subsequent to their conversion into the reparative MoMFs at the resolution phase of both CCl_4_-induced fibrosis (Figure 5A) and AILI (Figure 5B). The expression of membrane-bound MMP-14 by Ly6C^hi^ monocytes and MoMFs was especially intriguing, as most studies emphasize the importance of secreted MMPs like MMP-8,−9,−12, and−13 in reducing liver fibrosis (18, 19, 21–23). The enhanced co-localization of CX_3_CR1^+^ MoMFs with fibrotic collagen fibers as a consequence of GS341 treatment (Figure 4) suggests that MMP-14 under these conditions may obtain accessibility to degrade the scar tissue. Hence, we examined the expression pattern of MMP-14 in the fibrotic liver by performing mass cytometry (cyTOF) analysis at the peak of CCl_4_-induced fibrosis (48 h). Out of the total MMP-14^+^ cells in non-parenchymal enriched liver cells, ~90% were CX_3_CR1^+^ macrophages. In contrast, gating on MMP-9, another enzyme with collagenolytic properties, revealed that its expression mostly originates from neutrophils (~70%) but to some degree also from CX_3_CR1^+^ macrophages (20%) (Figure 5C). Flow cytometry analysis further confirmed the expression of MMP-14 mainly in MoMFs (defined as CD11b^+^Ly6G^−^CX_3_CR1^+^Ly6C^lo^ CD64^+^), while other innate immune cell populations present in the portal triad fibrotic area, such as Ly6C^hi^ monocytes and neutrophils, were mostly negative (Figure 5D). Interestingly, MoMFs from GS341-treated livers express higher levels of MMP-14 than those isolated from control Ab-treated livers (Figure 5E). Immunostaining of *Cx*_3_*cr1*^*gfp*/+^ liver sections further demonstrated increased accumulation of CX_3_CR1-GFP^+^ MoMF cells co-expressing MMP-14 in GS341-treated livers (Figure 5F). Recently, single-cell transcriptomic analysis of human liver in healthy and cirrhotic states uncovered the existence of scar-associated macrophages (SAMs), which were defined as analogous to MoMFs in mouse liver injury models. These cells, marked by the expression of TREM2 and CD9, expanded in the fibrotic niche and displayed a pro-fibrogenic phenotype (40). Data mining into their available gene expression dataset revealed the expression of MMP-14 among various parenchymal and non-parenchymal lineages mainly by mononuclear macrophages (MPs) and endothelial and epithelial cells. Among the MP populations, the main subpopulation expressing MMP-14 was SAMs (Figure S2). Collectively, these results outline that CX_3_CR1^+^ MoMFs express a myriad of secreted and membrane-bound collagen- and elastin-degrading enzymes.

### Ly6C^hi^ Monocytes and Their Monocyte-Derived Macrophage Descendants Drive Collagen Degradation in GS341-Treated Fibrotic Livers

**Figure 6:**
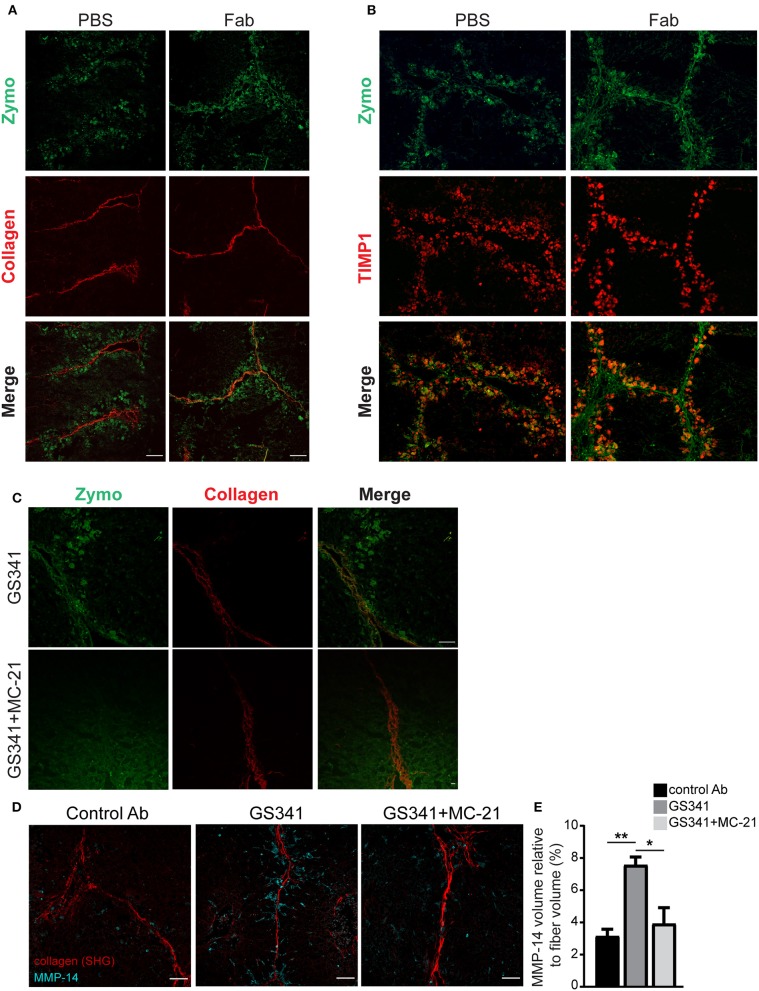
Ly6C^hi^ monocytes and monocyte-derived macrophages (MoMFs) facilitate collagen degradation following lysyl oxidase like 2 (LOXL2) inhibition. **(A)** Representative images of *in situ* zymography of fragment antigen binding (Fab)-treated and control livers 48 h after the last carbon tetrachloride (CCl_4_) injection. Spatial localization differences are demonstrated in collagen type I-degrading enzymes in Fab-treated livers compared to control [*n* = 5, green–zymography; red–second harmonic generation (SHG); scale−50 μm]. **(B)** Representative images of *in situ* zymography and tissue inhibitor of metalloproteinase (TIMP)1 staining of Fab-treated and control livers 48 h after the last CCl_4_ injection. **(C)** Representative images of *in situ* zymography of GS341– and GS341+ MC-21-treated mice 48 h after the last CCl_4_ injection (green–zymography; red–SHG; scale−50 μm). **(D)** Representative SHG images of 48 h liver samples treated with control antibody (Ab)/GS341/GS341+MC-21 Abs stained with MMP-14 antibody (scale−50 μm). **(E)** Quantification of the MMP-14 signal volume within 15 μm from the fiber, normalized to the total collagen fiber volume (*n* = 3). Data were analyzed by one-way ANOVA with Dunnett *post hoc* comparison. Results are presented as mean ± SEM with significance: *p < 0.05, **p < 0.01. Data in **(A–E)** represent a single experiment.

Given that Ly6C^hi^ monocytes and MoMFs express distinct collagenolytic MMPs (Figure 5), we conducted a spatially scar-resolving activity-based assay for collagen degradation by performing *in situ* zymography using fresh liver tissue sections. Using this technique, we monitored the localization of fibrillary collagen-degrading enzymes in liver sections of mice treated with the fragment antigen binding (Fab) moiety of GS341 or with control (PBS^−/−^) at the peak of the fibrotic phase (48 h). In the control group, collagenase activity (zymographic signal) exhibited minor co-localization with collagen fibers and was mostly distributed around them (Figure 6A). In sharp contrast, in the GS341-Fab-treated livers, collagenase activity co-localized with the collagen fibers (Figure 6A), resembling the collagenolytic activity displayed at the resolution phase of CCL_4_-induced liver fibrosis (120 h) (Figure 1D). Moreover, immunostaining for the endogenous MMP inhibitor TIMP1, a marker of liver fibrosis, together with zymographic analysis of collagenase activity demonstrated that GS341 treatment promotes the disengagement of collagenase activity from TIMP1, while moving toward the fibrotic scar area (Figure 6B). We could not detect any difference in the expression of TIMP1, TIMP2, or MMP-2 in whole liver lysates treated with GS341 or control Ab at 48 h (Figure S3). To further dissect the contribution of Ly6C^hi^ monocyte-derived MoMFs to this accelerated “on fiber” collagenase activity, we took advantage of the anti-CCR2 MC-21 antibody, which selectively depletes blood-circulating Ly6C^+^CCR2^+^ monocytes and thus prevents their infiltration to the injured liver (14, 38). Accordingly, in some of the GS341-treated mice, MC-21 was administered following the last CCl_4_ injection. Indeed, the inducible ablation of CX_3_CR1^+^ Ly6C^hi^ monocytes and their MoMF descendants eliminated the accelerated and augmented collagenolytic activity adjacent to, or on fibrotic fibers observed following LOXL2 inhibition (Figure 6C). Importantly, simultaneous imaging of both MMP-14 and collagenous matrix revealed higher expression of MMP-14 around the fibrillary collagen in the GS341-treated liver compared to the control, and these MMP-14-expressing cells were cleared by MC-21, confirming their Ly6C^hi^ monocyte ontogeny (Figure 6D). Further calculation of the vicinity of MMP-14 and the collagen fiber (up to a radius of 15 μm) revealed its higher proximity in the GS341-treated group, which was eliminated following MC-21-induced ablation of MoMFs (Figure 6E). Altogether, these results suggest that CX_3_CR1^+^ MoMFs gain greater accessibility to fibrotic fibers by virtue of LOXL2 inhibition, where they can use their unique repertoire of secreted and membrane-bound MMPs to degrade the fibrotic fibers.

## Discussion

Liver fibrosis-associated morbidity and mortality are progressively increasing worldwide, with no successful anti-fibrotic treatment available to date. Several reports implicate LOXL2 in ECM buildup and stabilization during liver fibrosis through crosslinking of structural proteins such as type I and III collagens and elastin (3, 6, 8–10). The development and accumulation of crosslinked fibers stabilize the fibrotic scar produced during the process of liver injury, rendering it more stable and therefore more resistant to degradation by matrix-degrading enzymes. Liver macrophages are very plastic and display opposing functions during liver fibrosis. A great deal of research has been invested in trying to prevent their pro-fibrotic behavior, while other therapeutic approaches have focused on elucidating switches that can promote and augment their reparative activity (26). Yet, with the progression of liver fibrosis, macrophages fail to resolve the formed scar tissue. The results presented here generate a direct link between LOXL2-governed ECM crosslinking and the impaired accessibility and scar-degrading activity of Ly6C^hi^ monocyte-derived MoMFs in the context of liver fibrosis. In this respect, therapeutic inhibition of extracellular LOXL2 activity in livers with preestablished fibrosis interferes with collagen packaging, rendering it more accessible to infiltrating Ly6C^hi^ monocyte-derived MoMFs, which uniquely bring to the fibrotic scar a set of membrane-bound and secreted collagen- and elastin-degrading enzymes. We especially outline here the provision by these cells of MMP-14 collagenase, which as being membrane tethered, depends upon the proximity and accessibility to collagen fibers.

Studies trying to understand the biochemical changes affecting fibrosis irreversibility have pinpointed LOXL2 as a key fibrogenic enzyme in liver fibrosis of various etiologies (3, 6, 8–10). In one study, the benefit of concurrent treatment with an allosteric anti-LOXL2 mAb (AB0023) in a reversible model of CCL_4_-induced liver fibrosis was assessed (9). AB0023 improved mouse survival and significantly reduced portoportal and portocentral bridging fibrosis, as well as the representation of α-SMA^+^ myofibroblasts in the portoportal septa (9). Yet, it remained unclear whether delayed LOXL2 neutralization would be as effective in the settings of preestablished biliary and non-biliary fibrosis. In this respect, in a follow-up study (12), delayed AB0023 treatment during progression (7–12 weeks) of thioacetamide (TAA)-induced fibrosis significantly reduced histological signs of bridging fibrosis. AB0023 treatment also promoted fibrosis reversal, with enhanced splitting and thinning of fibrotic septa, and a 45% decrease in collagen area 4 weeks after recovery from established TAA fibrosis (12). Furthermore, in two mouse models of biliary fibrosis, AB0023 similarly achieved significant anti-fibrotic efficacy and suppressed the ductular reaction, while hepatocyte replication increased (12).

Although these studies clearly associate between LOXL2 inhibition and reduction in hepatic collagen levels, mechanistic comprehension of this anti-fibrotic effect remains largely elusive. On one hand, LOXL2 may perpetuate fibrotic responses via HSC activation. Supporting this is the finding that exposure of both HSCs and portal fibroblasts in culture to increased mechanical tension on artificial polyacrylamide promotes their transition to myofibroblasts (41). Moreover, increased liver stiffness as a result of LOX-governed collagen crosslinking precedes fibrosis and potentially drives the HSC-myofibroblast transition (42). An additional mechanism was offered in an elegant study by the Popov group showing that blockage of LOXL2 by AB0023 can directly promote the differentiation of EpCAM^+^ hepatic progenitor cells (HPCs) toward regenerative hepatocytes, rather than fibrogenic ductal cell lineage commitment (12). LOXL2 expression by HPCs further suggests possible intracellular effects for this enzyme in determining the differentiation fates of these cells. Moreover, conditioned medium from HSC cultures promoted the growth of K19^+^ cholangiocytes from HPC cells and was likewise inhibited by AB0023. Therefore, the authors suggested that in the setting of fibrosis, autocrine/paracrine LOXL2 may favor HPC differentiation toward fibrogenic cholangiocytes. We present here an additional disease-modifying scheme. We show that LOXL2-governed collagen crosslinking limits scar degradation by liver-infiltrating, scar-destroying MoMFs. Previously, our lab demonstrated a role for LOXL2 in mediating morphological changes to collagen fibers *in vitro* and *in vivo* in a breast cancer model (29). Fibers grown in the presence of our novel active site anti-LOXL2 mAb (GS341) grow thinner and lose their directionality. Here we show for the first time, using high-resolution imaging, that inhibition of LOXL2 under conditions of progressive liver fibrosis changes the spatial organization of the fibrotic fibers. The resultant loosening of collagen assembly paves the way for the arrival of CX_3_CR1^+^ Ly6C^hi^ monocyte-derived MoMFs, which can now degrade the unraveled collagen and facilitate liver regeneration.

A previous study performed in a model of TAA-induced experimental liver fibrosis has explored the specific contribution of LOX and LOXL2 to fibrotic matrix stabilization utilizing specific blocking mAbs (M64 and AB0023, respectively) (12). Of these, only LOXL2 inhibition reduced the levels of insoluble collagen, a marker for collagen crosslinking. Here, we have used advanced high-resolution SEM imaging to uncover the effects of LOXL2 inhibition on collagen assembly *in situ* in a different model of liver fibrosis. Our results indicate reduced collagen packaging following treatment with GS341, suggesting indeed that LOXL2 is a key mediator of collagen remodeling during experimental liver fibrosis.

The increased matrix rigidity, which presumably accompanies LOXL2-mediated collagen crosslinking, may have direct implications on the migration and behavior of macrophages. Macrophages express mechanosensors such as integrins that can sense the ECM in their vicinity and transmit force from the extracellular environment via their interaction with numerous cytoskeletal and signaling proteins (43). Moreover, collagen and its digestion products can act as chemotactic stimuli for monocytes and macrophages (44, 45). Macrophages also adapt their migration mode to the matrix architecture and the biophysical parameters of the collagenous matrix (46). For example, fibrillar type I collagen favors an amoeboid migration mode, while denser 3D collagen I matrices promote a mesenchymal migration mode of human monocyte-derived macrophages (47). Nevertheless, the effects of the fibrotic matrix in general, and LOXL2-mediated modification of collagenous matrix in particular, on liver Ly6C^hi^ monocytes and MoMF migration and intra-hepatic motility have so far been overlooked. We show here that inhibition of LOXL2 activity during progressive liver fibrosis leads to increased accumulation of MoMFs within the liver parenchyma, particularly in the vicinity (up to 15 μ) of fibrotic collagen fibrils. Some of these macrophages actually adopt the shape of the collagen fiber while wrapping it. MoMFs may be actively attracted to the liver due to cues emanating from the exposure of buried epitopes as a result of the morphological changes in collagen fibers inflicted by LOXL2 inhibition, which favors collagen degradation by utilizing a membrane-bound effective protease. Alternatively, the reduced stiffness of the tissue may allow their easier entry into the liver. These options warrant further in-depth elucidation. In addition, a dense collagenous matrix may also dictate the behavior of MoMFs. Indeed, monocytes and macrophages grown on a collagen-rich matrix exhibit increased proliferation and acquire an alternatively activated M2 polarization state (48–50). Our results demonstrate accelerated collagen-degrading activity by MoMFs following LOXL2 inhibition and specifically increased expression of MMP-14. Further studies are required to determine whether MoMFs can directly sense collagenous matrix and to characterize the molecular reprogramming they are subjected to in the absence vs. presence of LOXL2-governed collagen remodeling. Overall, these results suggest that extracellular LOXL2-governed collagen remodeling impedes the arrival of these reparative macrophages to the sites of fibrosis, providing a possible mechanistic explanation for the failure of these cells to resolve scar tissue in settings of advanced fibrosis.

Gene expression analyses supported by mass and flow cytometry data and immunostaining revealed here that Ly6C^hi^ monocytes acquire a unique repertoire of scar-degrading enzymes upon differentiating into reparative MoMFs. These include secreted MMPs, such as MMP-12 and−13. These results are in agreement with previous studies (18, 19, 21–24). Yet, we also show that CX_3_CR1^+^ MoMFs uniquely express the membrane-bound MMP-14 gene and protein and that its expression by MoMFs is increased by LOXL2 inhibition. This inhibition leads to increased proximity of MoMFs to fibrous collagen fibers, which may provide access for membrane-based MMP-14 to facilitate focal collagen proteolysis. In human cirrhotic livers, MMP-14 expression is profoundly elevated in comparison with normal liver tissue. It is also increased during experimental CCL_4_-induced fibrosis, and its expression persists at the resolution phase (51). Yet, the involvement of MMP-14 in the pathogenesis and resolution of liver fibrosis remains so far ambiguous. Studies in a model of liver ischemia–reperfusion injury have indicated a role for MMP-14 in facilitating macrophage infiltration into the injured liver via interactions with fibronectin (52). Therefore, there may be a connection between the increased representation of MoMFs in GS341-treated livers and their higher expression of MMP-14. MMP-14 may also participate in the activation of MMP-13 (53), which is expressed by MoMFs as well.

Emerging evidence indicates that various cells composing the fibrotic niche can produce matrix cross-linkers belonging to the LOX family. Originally, HSCs and portal fibroblasts were noted as the major sources of LOXL1, LOXL2, and LOXL3. LOXL4 is widely expressed, while LOX in the normal liver appeared to be expressed primarily by hepatocytes and portal fibroblasts (6). As mentioned before, HPC cells can also produce LOXL2 and react to it in an autocrine and paracrine manner (12). Using immunoprecipitation assays, we show that GS341 specifically binds LOXL2. Yet, GS341 treatment reduces the protein expression levels of both LOXL2 and LOX collagen cross-linkers. Further studies are required to explain how inhibition of LOXL2 activity downregulates the production of LOX. In a recent study, single cell RNASeq transcriptomic profiling in human cirrhotic patients has actually detected the expression of both LOX and LOXL2 in the CD34^+^PLVAP^+^VWA1^+^ endothelial cell (EC) population, which expands in cirrhotic liver tissue and was found to be restricted to the fibrotic niche (40). The fact that these cells also express the fractalkine chemokine CX_3_CL1 (40) argues for their possible interaction with CX_3_CR1^+^ MoMFs. Using the gene expression dataset in this study, we show here that the expression of MMP-14 in human cirrhotic livers is associated with SAMs among MPs but is also expressed by ECs and epithelial cells, all constituents of the fibrotic niche. These results highlight that better comprehension of the LOXL2 and MMP-14 producing cells within the fibrotic niche is important for elucidating their pathophysiological crosstalk in the cirrhotic liver. For example, one may argue that in the context of progressive fibrosis, LOXL2 production by ECs and/or myo-fibroblasts within the fibrotic niche turns the collagen scar less accessible for degradation by MMP-14 expressed on SAMs.

It is worth noting that the proteolytic activity of MMPs can be regulated at multiple levels, including transcription, conversion from zymogen to active enzyme, compartmentalization and restraining by endogenous inhibitors such as TIMPs. Therefore, when judging the pathophysiological relevance of MMP expression in MoMFs, their collagenase activity has to be verified. In this respect, by using a spatially resolving activity-based assay for collagen degradation, we identified that collagen type I-degrading enzymes, such as MMPs, are already produced and active during the course of fibrosis progression and at the fibrosis peak (24–48 h). Nevertheless, their activity was not localized to the collagen fibers but rather to cells around the fibrotic areas and portal triads. Only during the regeneration phase (72–120 h), the zymographic activity of collagen type I degradation co-localized with the fibrotic collagen fibers, i.e., the bridging fibrotic fibers. Importantly, we show that delayed LOXL2 inhibition during the course of CCL_4_-induced fibrosis is sufficient to accelerate the appearance of this regeneration-like phenotype of co-localization already at the peak of fibrosis (48 h), concomitantly with uncoupling from the endogenous MMP inhibitor TIMP1. This “on fiber” transition of collagenase activity is diminished by the inducible ablation of Ly6C^hi^ monocytes and their MoMF descendants. Therefore, these results highlight that MoMFs are the primary source for collagen-degrading enzymes following LOXL2 inhibition.

As mentioned above, preclinical studies with the allosteric anti-LOXL2 AB0023 mAb have proved its efficacy in ameliorating biliary and non-biliary fibrosis (9, 12). Unfortunately, a humanized IgG4 monoclonal antibody against LOXL2, Simtuzumab® (Gilead Sciences SA), has failed so far to achieve a significant clinical benefit in patients with idiopathic pulmonary fibrosis (54), NASH (55, 56), or primary sclerosing cholangitis (57). Various factors may explain this failure to translate the overall positive preclinical results to the clinic: first, rodent models exhibit higher reversibility of liver fibrosis than do humans, especially with respect to patients suffering from cirrhosis and portal hypertension. Second, other compensatory pathways can drive collagen crosslinking, including other LOX isoforms and tissue transglutaminases. Third, there may be genetic and epigenetic changes in human patients that affect fibrosis progression and regression. We demonstrate here the use of a novel anti-LOXL2 mAb that directly targets the catalytic site of this enzyme, but its translational potency remains elusive. Moreover, we provide a new mechanistic view of how LOXL2 inhibition encourages the arrival of reparative MoMFs. Therefore, it will be extremely important in the future to delineate the molecular programs that favor macrophage restorative vs. pathological activity in liver fibrosis. In addition, immunotherapies that aim to enhance MoMF levels and/or reparative activity are already being clinically pursued (58, 59) and could have a synergistic effect with the anti-LOXL2 mAb presented here on the recovery process from liver fibrosis.

## Data Availability Statement

All datasets generated for this study are included in the article/Supplementary Material.

## Ethics Statement

The animal study was reviewed and approved by The Weizmann Institute of Science animal care and use committee protocol# 33070117-2.

## Author Contributions

MK, TG, NA, CV, and ISa designed, performed, and analyzed all experiments and wrote the manuscript. MV substantially assisted MK and TG with some of the major *in vivo* experiments and gene expression analyses. EB is a liver pathologist, who has performed histopathological assessment. SH-L and ISo helped with image and data analyses.

### Conflict of Interest

The authors declare that the research was conducted in the absence of any commercial or financial relationships that could be construed as a potential conflict of interest.
