# Supplementary material for: LOXL2 Inhibition Paves the Way for Macrophage-Mediated Collagen Degradation in Liver Fibrosis
Source: Front Immunol. 2020 Mar 31;11:480. doi: 10.3389/fimmu.2020.00480 (PMC7136575; doi:10.3389/fimmu.2020.00480)
Supplement: Supplementary file 1 [file Data_Sheet_1.docx]

Supplementary Material

**LOXL2 inhibition paves the way for macrophage-mediated collagen degradation in liver fibrosis**

Mordehay Klepfish^1,†^, Tamar Gross^1,†^, Milena Vugman^2^, Nikolaos A. Afratis^1^, Sapir Havusha^1^, Eli Brazowski^2^, Inna Solomonov^1^, Chen Varol^2,3,†^ and Irit Sagi^1,†^.

^1^ Department of Biological Regulation, Weizmann Institute of Science, Rehovot 76100, Israel.

^2^ Research Center for Digestive Tract and Liver Diseases, Tel Aviv Sourasky Medical Center, Tel Aviv-Yafo, Israel
^3^ Department of Clinical Microbiology and Immunology, Sackler Faculty of Medicine, Tel Aviv University, Tel Aviv-Yafo, Israel

^†^ Equal contribution

**Running title:** LOXL2 disturbs macrophage collagenolytic activity (5 words)

**Keywords**: Liver macrophages, LOXL2, Liver fibrosis, matrix metalloproteinases (MMPs), matrix metalloproteinase-14 (MMP-14), monocyte-derived macrophages

**CORRESPONDENCE**

Prof. Irit Sagi

[Irit.Sagi@weizmann.ac.il](mailto:Irit.Sagi@weizmann.ac.il)

Dr. Chen Varol

chenv@tlvmc.gov.il

**Supplementary Figures**

**Figure S1**


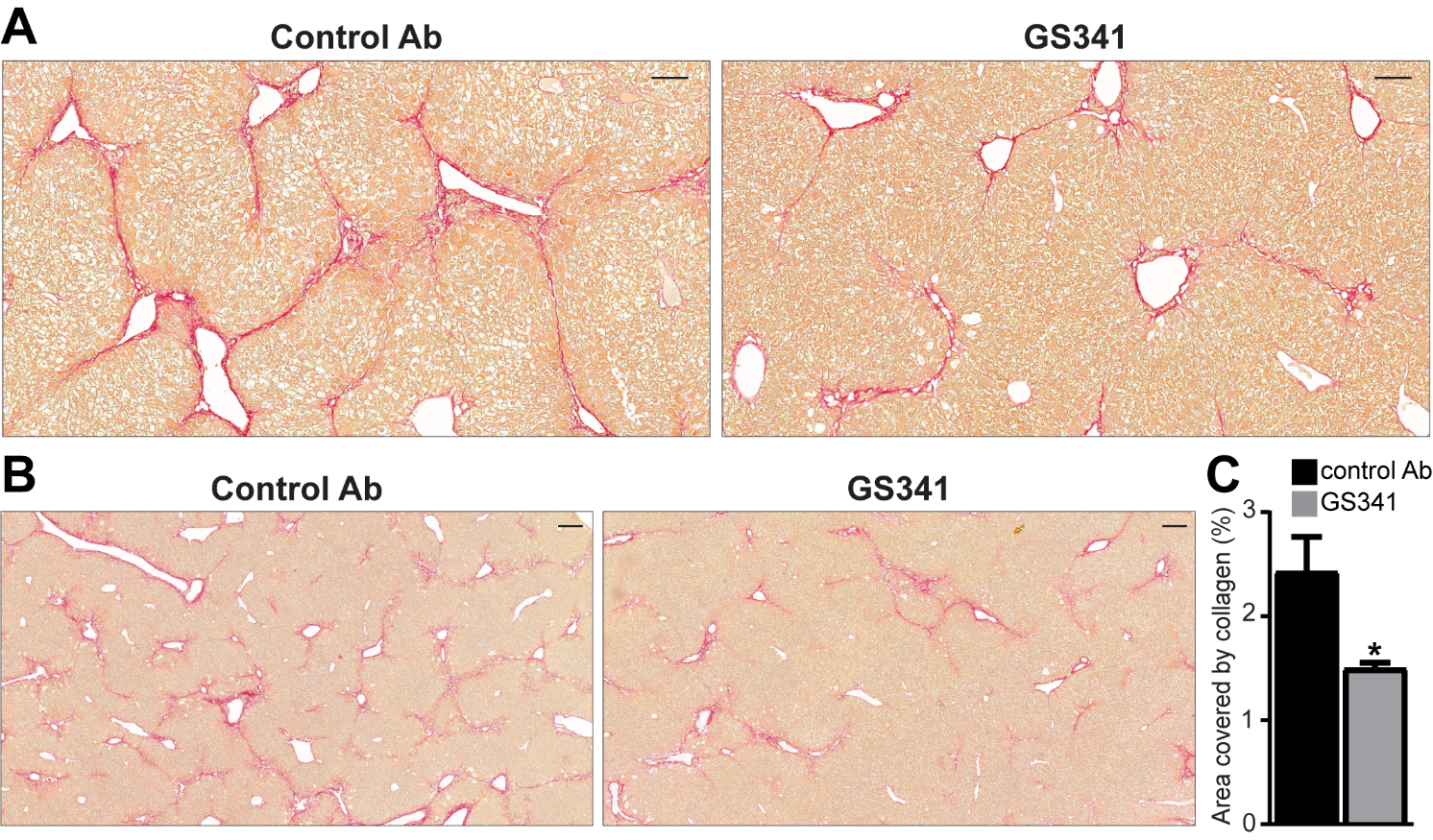


**Figure S1. GS341 attenuates CCl_4_-induced liver fibrosis at both 24 and 48 h following last CCL_4_-injection.**

**(A)** Representative images of Sirius red staining performed on paraffin-embedded slides of both GS341-treated and control Ab-treated livers excised 48 h after the last (9th) injection of CCl_4_ (n≥6; scale – 100 µm). **(B)** Representative images of Sirius red staining performed on paraffin-embedded slides of both GS341-treated and control Ab-treated livers excised 24 h after the last (9th) injection of CCl_4_ (n=5; scale – 200 µm). **(C)** Quantification of the percentage area covered by collagen, performed by ImageJ. Data was analyzed by an unpaired, two-tailed *t-test*. Results are presented as mean ± SEM with significance: *p<0.05. Data in panel B and C is a representative of a single experiment.

**Figure S2**

**
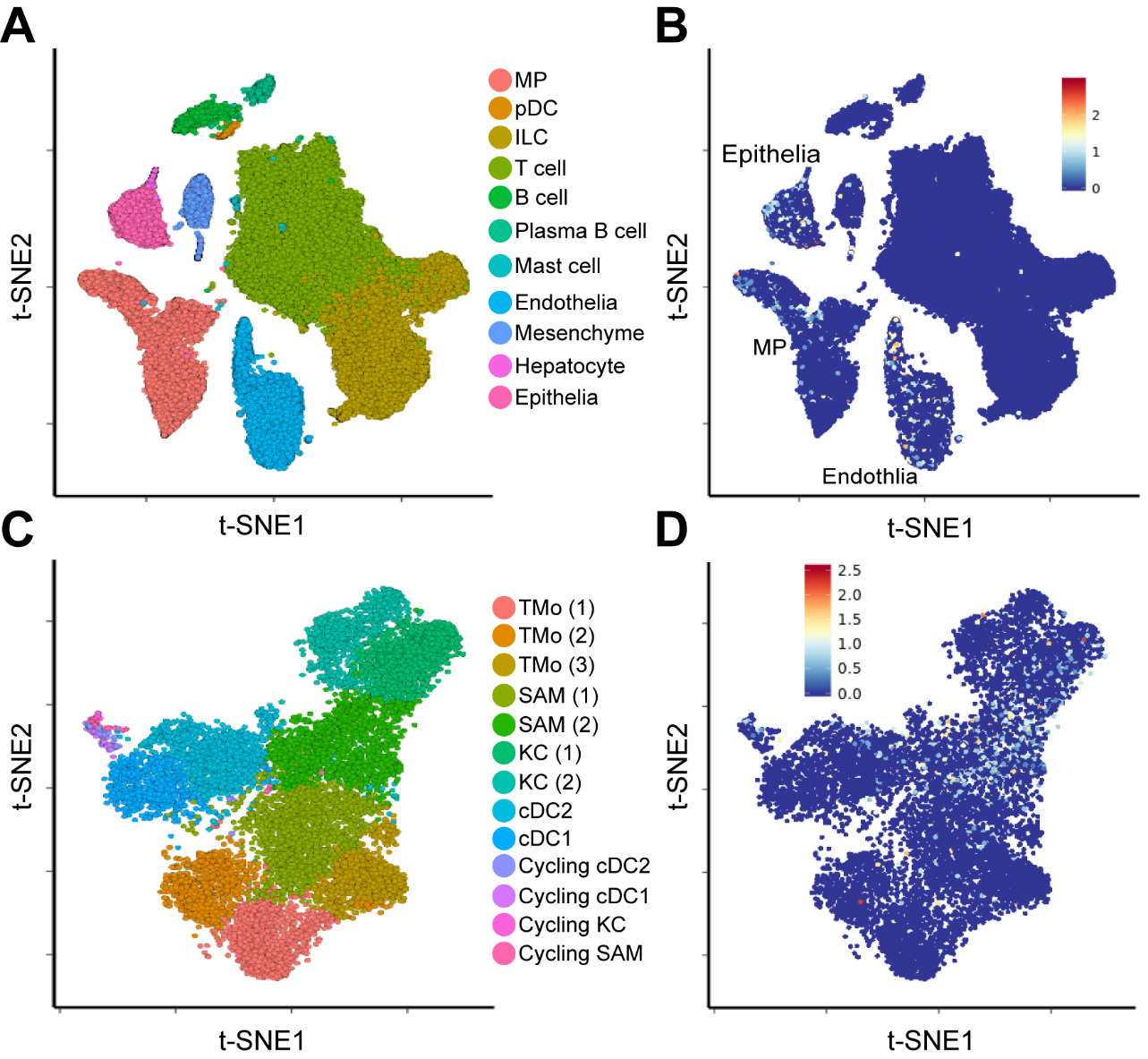
**

**Figure S2.** **MMP-14 cell origins in human liver cirrhosis**

The cell origin of MMP-14 in the liver was analysed from a transcriptome of single cells from human healthey and cirrotic livers [40]. **(A)** Cluster t-SNE of all cell lineages implied from expression of marker genes. ILC, innate lymphoid cell; MP, mononuclear phagocyte; pDC, plasmacytoid dendritic cell. **(B)** t-SNE of all cells lineage and the expression of MMP-14 among the cell populations. **(C)** Cluster t-SNE of mononucelar macrophages implied from expression of marker genes. **(D)** t-SNE of the mononuclear macrophages lineage and the expression of MMP14 among the cell populations. TMo, tissue monocyte; SAM, scar-associated macrophage; KC, kuffer cell; cDC, conventional dendritic cell. Data was extracted from the gene browser database ([http://www.livercellatlas.mvm.ed.ac.uk](http://www.livercellatlas.mvm.ed.ac.uk/), accession no. GSE136103).

**Figure S3**


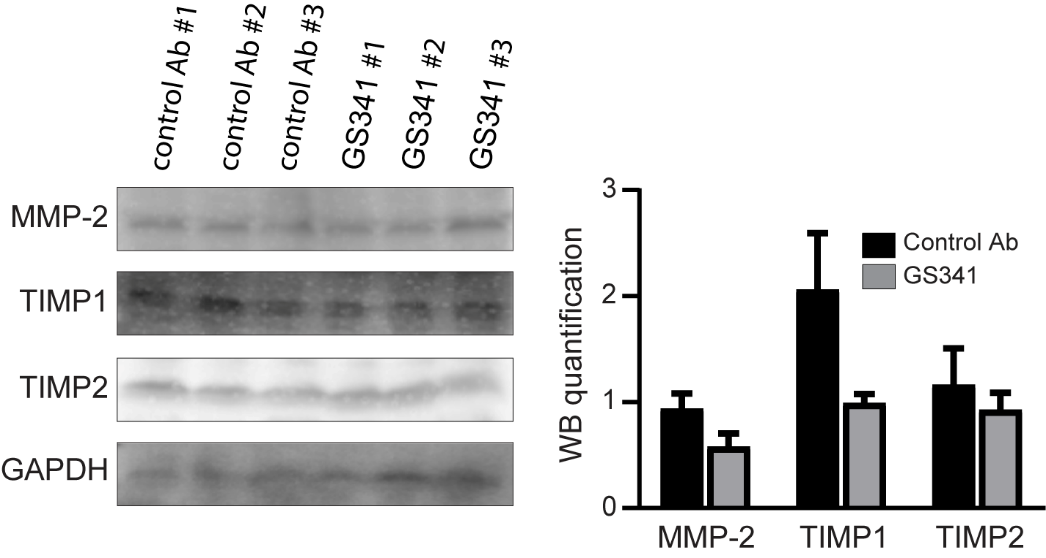


**Figure S3. Expression of MMP-2, TIMP1 and TIMP2 in whole livers of GS341- versus control Ab-treated mice**

Immunoblots stained with anti-MMP-2, anti-TIMP1 and anti TIMP2 antibodies of liver samples treated with control Ab or GS341, excised 48 h after the last CCl_4_ injection. The observed bend for MMP-2 is the active form (~63kD). The protein’s expression was normalized to GAPDH expression. Quantification was performed with ImageJ software.
